# Pharmacokinetics and pharmacodynamic effect of crenezumab on plasma and cerebrospinal fluid beta-amyloid in patients with mild-to-moderate Alzheimer’s disease

**DOI:** 10.1186/s13195-020-0580-2

**Published:** 2020-01-22

**Authors:** Kenta Yoshida, Anita Moein, Tobias Bittner, Susanne Ostrowitzki, Helen Lin, Lee Honigberg, Jin Y. Jin, Angelica Quartino

**Affiliations:** 1 Genentech, Inc., South San Francisco, CA 94080 USA; 2 F. Hoffmann-La Roche Ltd, Basel, Switzerland

**Keywords:** Pharmacokinetics, PK/PD, Plasma beta-amyloid, Nonlinear mixed-effects modeling, Target-mediated drug disposition model

## Abstract

**Background:**

Crenezumab, a fully humanized anti-beta-amyloid (Aβ) immunoglobulin G4 (IgG4) monoclonal antibody, binds to both monomeric and aggregated forms of Aβ. We assessed the pharmacokinetics (PK)/pharmacodynamics (PD) of crenezumab and its interaction with monomeric Aβ(1–40) and Aβ(1–42) peptides in serum/plasma and cerebrospinal fluid (CSF) samples from the phase II ABBY and BLAZE studies and the phase Ib GN29632 study.

**Methods:**

In ABBY, BLAZE, and GN29632 studies, patients with mild-to-moderate AD were treated with either placebo or crenezumab (300 mg subcutaneously every 2 weeks [q2w], or 15 mg/kg, 30 mg/kg, 45 mg/kg, 60 mg/kg, or 120 mg/kg intravenously q4w). Serum/plasma PK/PD analyses included samples from 131 patients who received crenezumab in all three studies. CSF PK/PD analyses included samples from 76 patients who received crenezumab in ABBY or BLAZE. The impact of baseline patient factors on Aβ profiles was also evaluated.

**Results:**

The serum concentration of crenezumab increased in a dose-proportional manner between 15 and 120 mg/kg q4w. Total monomeric plasma Aβ(1–40) and Aβ(1–42) levels significantly increased after crenezumab administration. The mean crenezumab CSF to serum ratio was ~ 0.3% and was similar across dosing cohorts/routes of administration. No clear correlation was observed between crenezumab concentration and Aβ(1–42) increase in CSF at week 69. The target-mediated drug disposition (TMDD) model described the observed plasma concentration–time profiles of crenezumab and Aβ well. Elimination clearance (CL_el_) and central volume of distribution (*V*_cent_) of crenezumab were estimated at 0.159 L/day and 2.89 L, respectively, corresponding to a half-life of ~ 20 days. Subcutaneous bioavailability was estimated at 66.2%.

**Conclusions:**

Crenezumab PK was dose proportional up to 120 mg/kg, with a half-life consistent with IgG monoclonal antibodies. Our findings provide evidence for peripheral target engagement in patients with mild-to-moderate AD. The study also showed that a model-based approach is useful in making inference on PK/PD relationship with unmeasured species such as free plasma Aβ levels.

**Trial registrations:**

ABBY: ClinicalTrials.gov, NCT01343966. Registered April 28, 2011. BLAZE: ClinicalTrials.gov, NCT01397578. Registered July 19, 2011. GN29632: ClinicalTrials.gov, NCT02353598. Registered February 3, 2015.

## Background

Alzheimer’s disease (AD) is the most common cause of dementia, thought to affect 47 million people worldwide [1]. Accumulations of beta-amyloid (Aβ) peptides and amyloid plaque deposition in the brain are characteristic of AD [2]. Aβ peptides can exist as monomers or aggregated forms (soluble oligomers, fibers, and plaque), and although the extent to which different Aβ species contribute to the pathophysiology of AD remains uncertain, in vitro and ex vivo evidence suggests that soluble oligomers may be major drivers of neurotoxicity [3–5].

Crenezumab is a fully humanized anti-Aβ immunoglobulin G4 (IgG4) monoclonal antibody (mAb) that binds to monomeric as well as aggregated forms of Aβ [6, 7]. In vitro, crenezumab has been shown to block Aβ aggregation, promote disaggregation, and protect neurons from oligomer-induced cytotoxicity [6]. Completed clinical trials of crenezumab in patients with mild-to-moderate AD include the phase II ABBY (NCT01343966) and BLAZE (NCT01397578) studies [8, 9]. Despite these studies not meeting their primary endpoints, exploratory post hoc analyses of the effects of crenezumab in a subset of patients with very mild AD in the high-dose 15 mg/kg intravenous (IV) cohort suggested the utility of testing earlier treatment of AD with higher doses of crenezumab [8]. Interim data from a phase Ib GN29632 study (NCT02353598) [10–12] supported further testing of higher doses of crenezumab. Two phase III studies (CREAD, NCT02670083; CREAD2, NCT03114657) investigated the efficacy and safety of crenezumab at a dose of 60 mg/kg IV every 4 weeks (q4w), i.e., fourfold higher than the high dose in phase II, compared with placebo in patients with early (prodromal-to-mild) AD [13, 14]. These studies were recently discontinued following a pre-planned interim analysis of CREAD, which indicated the study was unlikely to meet its primary endpoint; no safety signals were observed in this analysis and the overall safety profile was similar to that seen in previous trials [15]. A study of the efficacy and safety of crenezumab in individuals who carry the PSEN1 E280A autosomal-dominant mutation and do not meet the criteria for mild cognitive impairment due to AD or dementia due to AD and are, thus, in a preclinical phase of AD (autosomal-dominant AD) is ongoing [16].

Here we report the PK of crenezumab and its interaction with monomeric Aβ(1–40) and Aβ(1–42) peptides (peripheral target engagement pharmacodynamic (PD) biomarkers) as assessed in serum/plasma and cerebrospinal fluid (CSF) in samples from phase II ABBY and BLAZE studies and the phase Ib GN29632 study. Monomeric Aβ(1–40) and Aβ(1–42) peptides were evaluated in this analysis, as they can be measured reliably in plasma and CSF using automated high-precision immunoassays. While Aβ oligomers have also been measured successfully in CSF from the ABBY and BLAZE studies [17], it is technically challenging to measure those forms of Aβ in plasma. PK/PD characteristics of mAbs such as crenezumab, and their interactions with target molecules, can be described by target-mediated drug disposition (TMDD) models, which take into account the binding affinity of the antibody for its target molecule and the resulting degradation/clearance of the antibody–target complex and as such can provide insights to *unmeasured* species such as free target concentrations. This is of high value as development of assays for free targets are often technically challenging. We constructed a TMDD model to describe crenezumab serum concentrations and plasma Aβ(1–40) and Aβ(1–42) peptide levels in patients treated with crenezumab to help quantitatively interpret observed interactions and simulate the concentration of unmeasured species, such as free plasma Aβ. In addition, plasma Aβ levels have been reported to be influenced by baseline patient characteristics, e.g., age and renal function [18]; therefore, we also used this model to assess the impact of baseline patient characteristics on the Aβ profiles.

## Methods

### Study design and subjects

In this analysis, crenezumab PK and PD data, i.e., serum total crenezumab concentrations and plasma total monomeric Aβ(1–40) and Aβ(1–42) levels, collected from patients enrolled in the phase II ABBY and BLAZE studies and the phase Ib GN29632 study were used. The detailed methodology, study randomization, and sample size determination for the studies have been described previously (Table 1) [8, 9, 12].

**Table 1:** Overview of characteristics of included crenezumab studies

Study	Phase	Design	Treatments	Subjects
ABBY [8]	II	Double-blind, placebo-controlled, randomized, parallel-group study	*Part 1*: Low-dose 300 mg SC crenezumab q4w *Part 2:* High-dose 15 mg/kg IV crenezumab q4w Placebo q4w *SRI:* At least 2 monthly administrations of 15 mg/kg IV crenezumab or placebo	431 patients with mild-to-moderate AD aged 50–80 years were randomized 2:1 (crenezumab:placebo) - *Part 1*: 184 patients - *Part 2:* 241 patients - *SRI:* 13 patients
BLAZE [9]	II	Double-blind, placebo-controlled, randomized study	*Part 1*: Low-dose 300 mg SC crenezumab q4w *Part 2*: High-dose 15 mg/kg IV crenezumab q4w Placebo q4w	91 Aβ-positive patients with mild-to-moderate AD aged 50–80 years were randomized 2:1 (crenezumab:placebo) - *Part 1:* 39 patients - *Part 2:* 52 patients
GN29632 [10–12]	Ib	Double-blind, placebo-controlled, randomized study followed by open-label extension	Double-blind phase: *Cohort 1:* 30 or 45 mg/kg IV crenezumab q4w *Cohort 2:* 60 mg/kg IV crenezumab q4w *Cohort 3:* 120 mg/kg IV crenezumab q4w Placebo q4w Open-label extension: *Cohort 1 and 2* could continue to receive crenezumab at the originally assigned dose^a^ *Cohort 3* switched to 60 mg/kg q4w *Placebo* could cross over to crenezumab at the originally assigned dose and 60 mg/kg if assigned to cohort 1 or 3	75 patients with mild-to-moderate AD aged 50–90 years were randomized 5:1 at each of the crenezumab dosing levels, or placebo up to week 13: - *Cohort 1:* 30 mg/kg: 10 patients 45 mg/kg: 11 patients - *Cohort 2:* 21 patients - *Cohort 3:* 19 patients 71 patients entered the open-label extension

^a^Following a protocol amendment, patients in cohort 1 could increase to 60 mg/kg q4w dose after week 133. *Abbreviations: Aβ* beta-amyloid, *AD* Alzheimer’s disease, *IV* intravenous, *q4w* every 4 weeks, *SC* subcutaneous, *SRI* safety run-in

ABBY was a phase II, randomized, double-blind, placebo-controlled study designed to evaluate the safety and efficacy of crenezumab in patients with mild-to-moderate AD [8]. Patients received low-dose 300 mg SC crenezumab or placebo q2w, or high-dose 15 mg/kg IV crenezumab or placebo q4w. To assess the potential for using a higher dose of crenezumab compared with phase I, part 2 of ABBY was preceded by a safety run-in (SRI) period (for SRI dosing schemes, see Table 1) [8].

BLAZE was a phase II, randomized, double-blind, placebo-controlled study designed to evaluate the effects of crenezumab on brain amyloid plaque load as assessed by florbetapir positron emission tomography (PET) and other biomarkers in patients with mild-to-moderate AD [9]. Patients were required to have evidence of elevated amyloid burden consistent with a diagnosis of AD. The study was conducted in two parts as described above for the ABBY study without the SRI period/cohort. Dosing regimens and patient numbers for both ABBY and BLAZE are described in Table 1.

In both ABBY (including the SRI period) and BLAZE, blood samples were collected for PK measurement of serum crenezumab concentrations at week 1 (on day 1, including a pre-dose baseline sample), at specified times throughout the treatment period (through week 69), during the efficacy and safety follow-up visits (weeks 73, 81, and 85), and at end-of-treatment/treatment discontinuation visits. For patients in the SC cohort, samples were collected prior to study drug administration on dosing days; for those in the IV cohort, samples were collected prior to study drug administration and 60 min after the end of infusion, unless otherwise specified. Blood samples for PD analysis were collected during the screening period and at the week 1 (day 1) visit, at specified times during the treatment period (through week 69), weeks 73, 81, and 85 of the safety follow-up visits, and at end-of-treatment/treatment discontinuation visits. On dosing days, blood samples for PD analysis were collected prior to study drug administration. In ABBY, CSF samples were collected as an optional procedure at week 1 (day 1/baseline) and before study drug administration at week 69 (steady state). In BLAZE, CSF samples were collected from all patients at screening and prior to dosing at week 69 or at early termination/discontinuation, if necessary.

GN29632 was a phase Ib, multicenter, randomized, double-blind, placebo-controlled, parallel-group ascending dose study followed by an open-label extension (OLE) study. This study was designed to assess the safety, tolerability, and PK of crenezumab delivered at higher doses than those used in the phase II program. Patients were randomly assigned to one of the three cohorts and received between 30 and 120 mg/kg IV q4w of crenezumab or placebo. Dosing regimens and patient numbers per cohort are detailed in Table 1. Blood samples for serum PK and plasma PD analyses were collected at baseline (week 1/day 1; pre-dose, 1 min, and 60–90 min post-administration), week 1/day 2, week 1/day 8 (± 2 days), week 2/day 15 (± 2 days), week 5 (± 2 days), week 9 (± 2 days), week 13 (± 2 days), week 21 (± 2 days), at end of treatment, and at the start of the OLE study. Per protocol, all scheduled serum PK and plasma PD samples were obtained just prior to study drug administration and 60–90 min after the end of infusion.

All studies were conducted in accordance with the ethical principles of the Declaration of Helsinki and complied with Good Clinical Practice. A central investigational review board and individual site institutional review boards reviewed and provided approval for the protocols as well as informed consent forms. All subjects provided informed consent and consent for publication.

### PK/PD assessments

Total serum crenezumab concentrations were analyzed using a validated enzyme-linked immunosorbent assay with a lower limit of quantification of 0.05 μg/mL for serum and 0.012 μg/mL for CSF. Total monomeric plasma Aβ(1–40) and Aβ(1–42) levels (which correspond to free and crenezumab-bound Aβ levels) were measured using a robust, non-commercial Elecsys® drug-tolerant prototype assay on the cobas® e411 analyzer (Roche Diagnostics, Rotkreuz, Switzerland), with a lower limit of detection of < 2 pg/mL (both assays). For the ABBY study, we measured Aβ levels only for the SRI cohort with the current assay system due to method availability, and only the data from that cohort (in addition to data from BLAZE and phase Ib studies) are included in subsequent analyses. Total CSF crenezumab concentrations were analyzed using a validated enzyme-linked immunosorbent assay (limit of detection 12.5 ng/mL) as described previously [9]. Total Aβ(1–42) in CSF was measured using the Elecsys® β-Amyloid (1–42) immunoassay commercially available from Roche Diagnostics (Penzberg, Germany), which was confirmed to be tolerant to the presence of crenezumab in the sample [19].

### PK/PD analysis

Crenezumab PK and Aβ kinetics from all randomized subjects who received at least one dose of study treatment (placebo or active) and had at least one post-dose assessment of both PK and Aβ levels during the three studies were analyzed using nonlinear mixed-effects modeling with NONMEM (version 7.3., ICON Development Solutions, Ellicott City, MD, USA). In total, 1332 serum PK and 2203 plasma PD samples from 131 patients were used for the analysis.

**Fig. 1:**
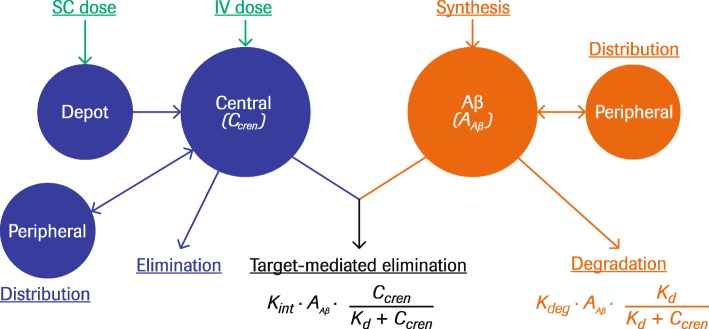
Schematic representation of the TMDD model structure. According to Michaelis–Menten approximation, the drug–target complex is expected to be in quasi-equilibrium with the concentration of monoclonal antibody > > target concentration. *Abbreviations: A*_*Aβ*_ amount of beta-amyloid, *Aβ* beta-amyloid, *C*_*cren*_ concentration of crenezumab, *IV* intravenous, *K*_*d*_ equilibrium constant governing antibody-ligand binding, *k*_*deg*_ first-order rate constant for free ligand degradation, *k*_*int*_ zero-order input rate constant for ligand, *SC* subcutaneous, *TMDD* target-mediated drug disposition

A TMDD model with Michaelis–Menten approximation [20] was used to describe the observed serum concentrations of crenezumab, plasma concentrations of Aβ(1–40) and Aβ(1–42), and the PK/PD relationship (Fig. 1). Michaelis–Menten approximation was selected because crenezumab concentration is in excess of the Aβ concentration at clinical doses and Aβ concentration increases upon crenezumab administration [20].

A stepwise covariate model (SCM) building tool developed in Perl-speaks-NONMEM (PsN), version 4.7.0 [21] was used to assess the impact of baseline patient factors, including age at enrollment, sex, body weight, and glomerular filtration rate (GFR) on Aβ profiles, with *p* values of 0.01 and 0.005 as criteria for the forward selection and backward elimination, respectively.

For evaluating PK/PD relationships in CSF, 76 PK and 74 PD samples from 76 patients enrolled in ABBY and BLAZE studies and receiving active treatment were analyzed using descriptive statistics.

## Results

### Serum crenezumab concentration and plasma Aβ(1–40) and Aβ(1–42) levels after the first dose of crenezumab

**Fig. 2:**
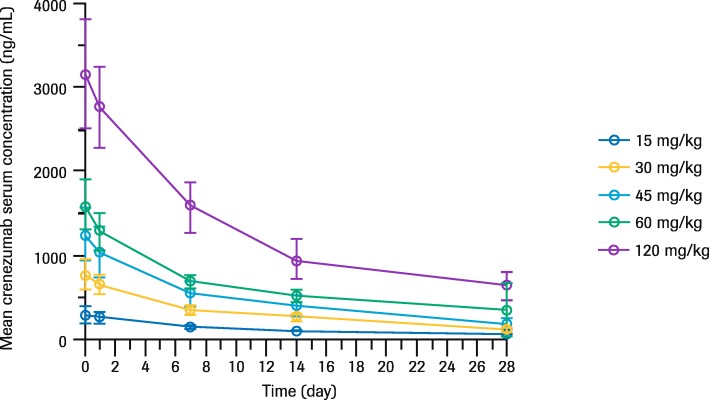
Mean (SD) serum crenezumab concentrations after initial dose (weeks 1–5). Each line represents mean crenezumab serum concentration following IV administration; doses of 15 mg/kg (ABBY; BLAZE) and 30–120 mg/kg (GN29632 phase Ib study). *Abbreviations: IV* intravenous, *SD* standard deviation

The serum PK profile of crenezumab showed a biphasic disposition over the 28-day period following the first dose (Fig. 2). The serum concentration of crenezumab increased in a dose-proportional manner between 15 and 120 mg/kg q4w doses.

**Fig. 3:**
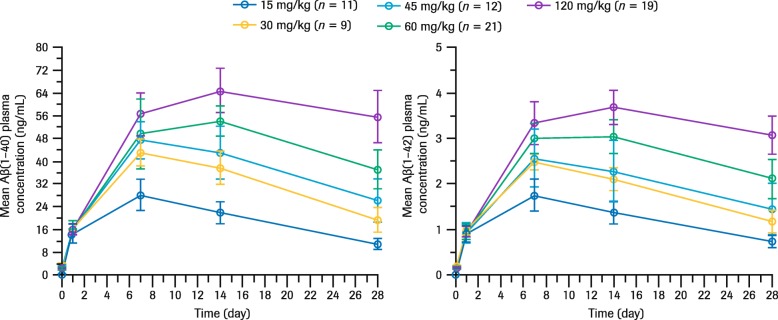
Mean (SD) total plasma Aβ(1–40) and Aβ(1–42) concentrations after initial dose (weeks 1–5). Total number of patients included = 72. Each line represents mean total Aβ(1–40) or Aβ(1–42) plasma concentration following IV administration; data shown are from the phase II ABBY study (SRI cohort) for 15 mg/kg dose and phase Ib GN29632 for 30–120 mg/kg doses. *Abbreviations: Aβ* beta-amyloid, *IV* intravenous, *SD* standard deviation, *SRI* safety run-in

**Fig. 4:**
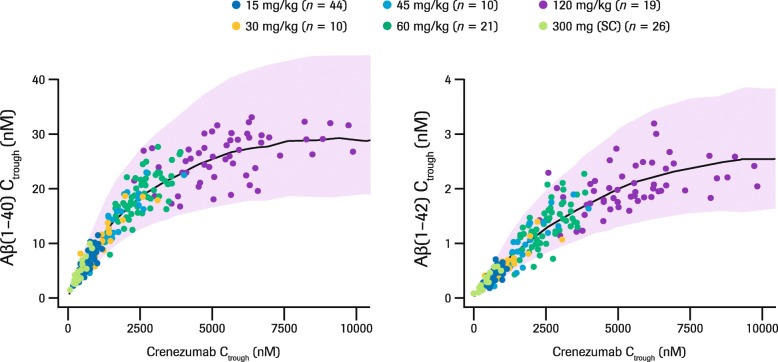
Serum crenezumab and total plasma Aβ C_trough_ concentrations. Total number of patients included = 130. Circles represent data from the phase II ABBY and BLAZE studies for all patients for 300 mg q2w SC and 15 mg/kg q4w IV, and from the phase Ib GN29632 study for 30–120 mg/kg q4w IV from weeks 5, 9, and 13. Solid lines and shaded areas represent the median and 5–95% prediction intervals, respectively, based on target-mediated drug disposition model predictions. *Abbreviations: Aβ* beta-amyloid, *C*_*trough*_ pre-dose serum crenezumab, *IV* intravenous, *q2w* every 2 weeks, *q4w* every 4 weeks, *SC* subcutaneous

Total monomeric plasma Aβ(1–40) and Aβ(1–42) levels significantly increased after administration of crenezumab, demonstrating peripheral target engagement (Fig. 3). PD response was delayed compared with the time to peak crenezumab concentration and reached its maximum level 7–14 days after the initial dose (Fig. 3). Total plasma Aβ levels increased in a dose-dependent, but not dose-proportional manner. Analyses of pre-dose serum crenezumab (*C*_trough_) and plasma Aβ suggest that the accumulation of total plasma Aβ reached a plateau following crenezumab 120 mg/kg q4w dosing (Fig. 4).

### CSF crenezumab concentrations and CSF Aβ(1–42) levels

The ratio of crenezumab detected in the CSF vs serum was consistent across dosing cohorts/routes of administration with a mean crenezumab CSF to serum ratio of ~ 0.3%. The mean (standard deviation) steady-state pre-dose crenezumab concentrations in CSF were low (0.19 [0.14] μg/mL in the 300 mg SC cohort; 0.25 [0.12] μg/mL in the 15 mg/kg IV cohorts). As described previously for the BLAZE study [9], there was a significant increase in CSF total Aβ(1–42) concentrations in patients treated with crenezumab. In the low-dose SC cohort, CSF Aβ(1–42) mean change from baseline was − 52.11 pg/mL in the placebo arm, while in the crenezumab arm mean change from baseline was + 74.90 pg/mL (crenezumab vs placebo difference of 127.01 pg/mL, *p* = 0.001). In the high-dose IV cohort, CSF Aβ(1–42) mean change from baseline was − 86.65 pg/mL in the placebo arm, whereas in the crenezumab arm mean change from baseline was + 7.86 pg/mL (crenezumab vs placebo difference of 94.51 pg/mL, *p* = 0.022).

**Fig. 5:**
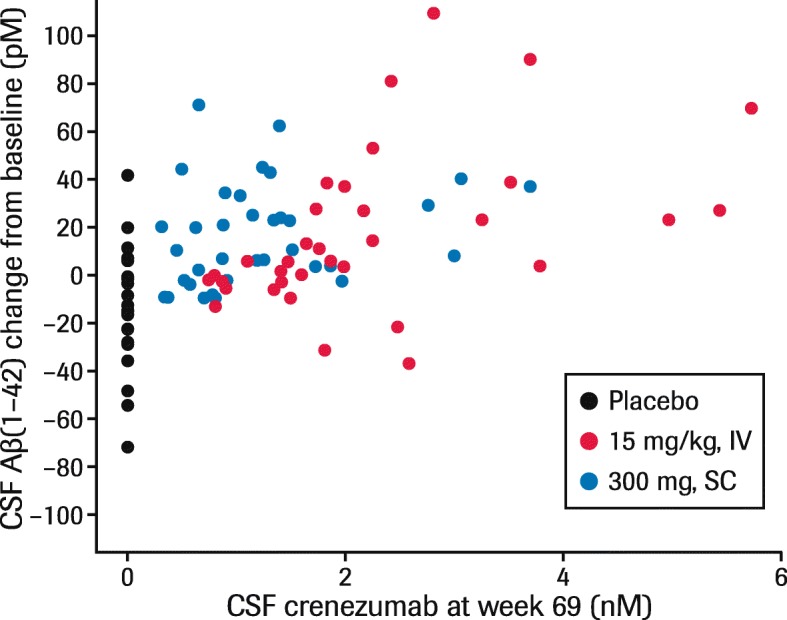
Steady-state pre-dose crenezumab concentration and Aβ(1–42) change from baseline in CSF at week 69. Data shown are from the phase II ABBY and BLAZE studies for patients enrolled in 300 mg q2w SC or 15 mg/kg q4w IV cohorts. *Abbreviations: Aβ* beta-amyloid, *CSF* cerebrospinal fluid, *IV* intravenous, *q2w* every 2 weeks, *q4w* every 4 weeks, *SC* subcutaneous

There was no clear PK/PD correlation between steady-state pre-dose CSF crenezumab levels and CSF Aβ(1–42) changes at week 69 (Fig. 5). The crenezumab serum and CSF concentrations in BLAZE were similar to those in ABBY [8, 9].

### Model-based analysis of serum crenezumab and plasma Aβ(1–40) and Aβ(1–42) concentrations

**Fig. 6:**
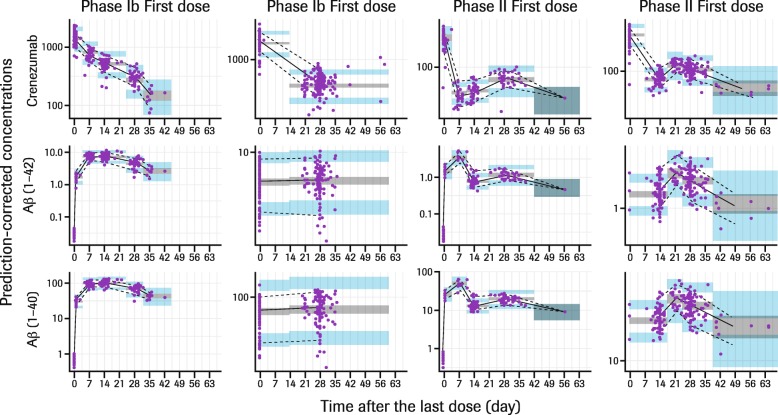
Prediction-corrected visual predictive check plots. Phase Ib, data from GN29632 study; phase II, data from ABBY and BLAZE studies. Circles represent observed data. Solid and dashed lines represent median and 90th percentiles of observed data, respectively. Gray and blue shaded areas represent simulated 90% prediction interval of median and 90th percentiles, respectively. *Abbreviations: Aβ* beta-amyloid, *Ph* phase

The final TMDD model with Michaelis–Menten approximation (Fig. 1) described the observed profiles of crenezumab and Aβ well (Fig. 6). Overall, the PK/PD parameter values obtained through the nonlinear mixed-effects modeling were reasonable, with relative standard error of less than 10% for most fixed effects (Table 2). Estimated baseline plasma Aβ(1–40) and Aβ(1–42) values were 142 pM and 5.98 pM, respectively, and between-subject variability (BSV) values were 8.3% and 13.8%, respectively. The estimated equilibrium constant governing antibody-ligand binding (*K*_d_) levels for Aβ(1–40) and Aβ(1–42) were 12 nM and 9.37 nM, respectively. Crenezumab exhibited dose-proportional PK with the estimated elimination clearance (CL_el_) and central volume of distribution (*V*_cent_) of 0.159 L/day and 2.89 L, respectively, which are consistent with typical values for IgG mAbs [22] and correspond to a half-life (*t*_1/2_) of ~ 20 days. SC bioavailability (*F*_sc_) was estimated at 66.2%. Estimated intrinsic clearance of the crenezumab–Aβ complex (CL_int_) was 1.01 L/day, sixfold faster than that of crenezumab alone (Table 2).

**Table 2:** Estimated PK/PD parameters determined using nonlinear mixed-effects modeling

Parameter	Estimate	RSE (%)	BSV (CV%)	RSE (%)
Crenezumab				
CL_el_ (L/day)	0.159	3.0	19.1	8.4
CL_int_ (L/day)	1.01	5.8	15.3	25
*V*_cent_ (L)	2.89	3.4	18.3	8.8
*V*_periph_ (L)	1.84	10	75.2	9.7
*Q* (L/day)	0.142	4.8	–	–
*F*_sc_ (%)	66.2	3.9	–	–
*K*_a_ (/day)	0.161	6.4	–	–
*K*_d_ Aβ(1–40) (nM)	12.0	3.0	13.6^a^	12
*K*_d_ Aβ(1–42) (nM)	9.37	2.5	13.6^a^	12
BWT (kg) on CL_el_	0.835	12	–	–
BWT (kg) on CL_int_	0.474	42	–	–
BWT (kg) on *V*_cent_	0.725	17	–	–
Aβ(1–40)				
Baseline (pM)	142	1.3	8.3	10
*K*_deg_ (/day)	114	5.7	17.9^a^	12
*Q* (L/day)	8.72	31	–	–
*V*_periph_ (L)	3.77	11	–	–
Age (year) on baseline	0.0037	27		
GFR (mL/min/1.73 m^2^) on baseline	− 0.0035	20		
Sex (male) on *K*_deg_^b^	− 0.16	36		
Aβ(1–42)				
Baseline (pM)	5.98	1.7	13.8	9.9
*K*_deg_ (/day)	287	6.5	17.9^a^	12
*Q* (L/day)	229	15	–	–
*V*_periph_ (L)	8.19	7.8	–	–
GFR (mL/min/1.73 m^2^) on baseline	− 0.0048	22		
Sex (male) on *K*_deg_^b^	− 0.16	36		

Patient factors are incorporated as the exponential and linear form on the parameters for crenezumab and Aβ, respectively. ^a^Same BSV was used on Aβ(1–40) and Aβ(1–42) for *K*_d_ and *K*_deg_. ^b^The same coefficient was used on Aβ(1–40) and Aβ(1–42) for covariate effect of sex on *K*_deg_. *Abbreviations: Aβ* beta-amyloid, *BSV* between-subject variability, *BWT* body weight, *CL*_*el*_ elimination clearance (of crenezumab), *CL*_*int*_ intrinsic clearance (of crenezumab–Aβ complex), *CV* coefficient of variation, *F*_*SC*_ subcutaneous bioavailability, *GFR* glomerular filtration rate, *K*_*a*_ absorption rate constant, *K*_*d*_ equilibrium constant governing antibody-ligand binding, *K*_*deg*_ degradation rate constant, *PD* pharmacodynamics, *PK* pharmacokinetics, *Q* inter-compartment clearance, *RSE* relative standard error, *V*_*cent*_ central volume of distribution, *V*_*periph*_ peripheral distribution volume

### Analysis of impact of patient baseline characteristics on crenezumab and Aβ kinetics in plasma and serum

**Fig. 7:**
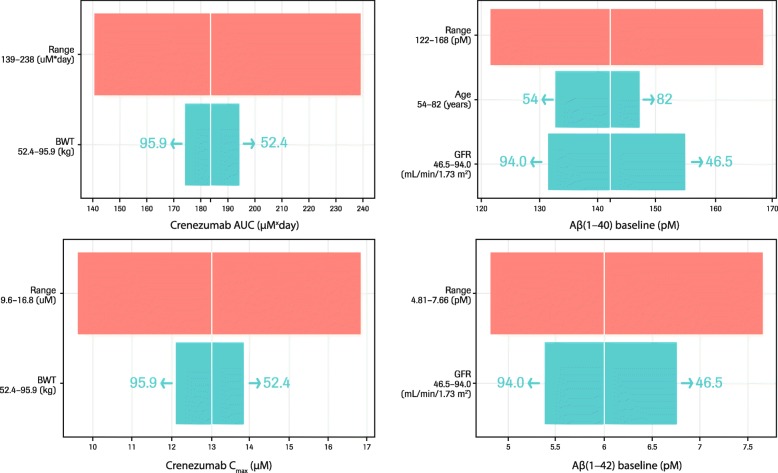
Predicted impact of patient baseline characteristics on PK profile of crenezumab and plasma Aβ levels. White vertical line refers to the predicted parameters for a 72-year-old male, weighing 72.3 kg, with a GFR of 72.5 mL/min/1.73 m^2^ after a crenezumab 60 mg/kg IV dose. Red bar depicts the 5th–95th percentile crenezumab exposure (left column) or Aβ levels (right column) range across the entire population. Green bar represents the influence of a single covariate on the predicted parameters. The upper and lower values for each covariate represent 90% of the observed covariate range in the population. *Abbreviations: Aβ* beta-amyloid, *AUC* area under the curve, *BWT* body weight, *C*_*max*_ peak concentration, *GFR* glomerular filtration rate, *IV* intravenous, *PK* pharmacokinetics

We evaluated the effects of selected patient characteristics (subsequently referred to as covariates) on crenezumab and plasma Aβ kinetics. Body weight was a statistically significant covariate for crenezumab PK (CL_el_, CL_int_, and *V*_cent_), age and GFR were statistically significant covariates for baseline plasma Aβ levels, and sex was a statistically significant covariate for degradation rate constant of plasma Aβ (*K*_deg_). Estimated BSV of CL_el_ and *V*_cent_ decreased from 25 to 19% and 23 to 18%, respectively, after incorporating the effect of body weight, although it did not appear to be a major source of the observed BSV; between 90% of the observed range (52.4–95.9 kg), differences in area under the curve (AUC) and peak concentration (*C*_max_) were 1.1-fold higher (higher exposure with smaller body weight) (Fig. 7). After incorporating the effect of age and GFR, estimated BSV for baseline levels of Aβ(1–40) and Aβ(1–42) decreased from 11 to 8.3% and 16 to 14%, respectively. Differences in baseline levels of Aβ(1–40) and Aβ(1–42) in 90% of the observed range of GFR (94.0 to 46.5 mL/min/1.73 m^2^) were 1.2- and 1.25-fold higher in patients with smaller GFR. Differences in baseline levels of Aβ(1–40) in 90% of the observed range of age (54–82 years) were 1.1-fold higher in patients with higher age (Fig. 7). Incorporating the effect of sex on *K*_deg_ decreased BSV from 19 to 18%.

### Simulations of the effect of varying crenezumab doses on free and bound Aβ kinetics based on the developed PK/PD model

**Fig. 8:**
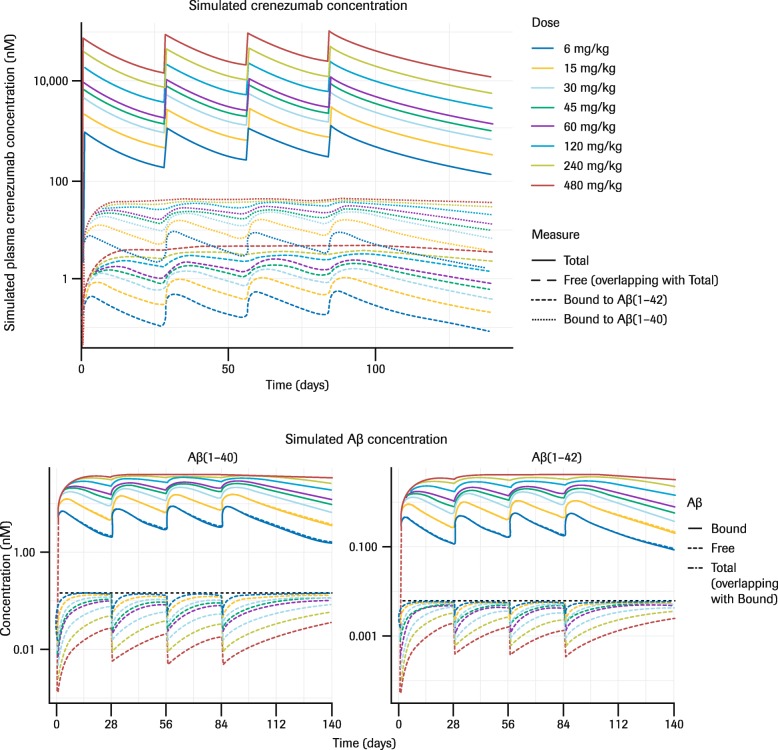
Simulations illustrating effects of varying crenezumab doses (mg/kg q4w) on plasma Aβ kinetics based on the developed PK/PD model. Note that total and free crenezumab concentrations overlap with each other. *Abbreviations: Aβ* beta-amyloid, *PD* pharmacodynamics, *PK* pharmacokinetics, *q4w* every 4 weeks

Crenezumab was detected in a large excess compared with plasma Aβ levels (approximately 150-fold difference at steady-state C_trough_ for 60 mg/kg dose); therefore, the crenezumab in serum was predominantly free. As may therefore be expected, the modeling data suggested that plasma Aβ was predominantly bound (Fig. 8). The reduction in free plasma Aβ levels associated with crenezumab treatment was better maintained at higher doses, even after the increase in total Aβ level had plateaued.

## Discussion

In this pooled analysis of data from the phase II ABBY and BLAZE studies and the phase Ib GN29632 study, we showed that the PK of crenezumab in patients with mild-to-moderate AD was dose proportional across the dose range tested (15–120 mg/kg q4w IV and 300 mg q2w SC) and was characterized by clearance and *t*_1/2_ values expected of IgG mAbs (0.159 L/day and *t*_1/2_ ~ 20 days, respectively) (Table 2) [22]. Body weight was shown to influence the elimination clearance of crenezumab (CL_el_), intrinsic clearance of crenezumab–Aβ complex (CL_int_), and central distribution volume (*V*_cent_), all of which increased with increasing body weight (Fig. 7). These findings are consistent with the results observed for other therapeutic IgG molecules that exhibit linear kinetics in humans [22].

Total plasma Aβ(1–40) and Aβ(1–42) levels increased significantly following each administration of crenezumab demonstrating peripheral target engagement of monomeric Aβ(1–40) and Aβ(1–42). The increase in total Aβ(1–40) and Aβ(1–42) levels can be attributed to slower elimination of crenezumab–Aβ complex than free Aβ(1–40) and Aβ(1–42): 0.36 vs 114 [Aβ(1–40)] or 287 [Aβ(1–42)] on day 1. The observed increase in total plasma Aβ levels was dose dependent but not dose proportional and reached a plateau with the 120 mg/kg q4w IV dose. A TMDD model developed to characterize the observed serum crenezumab concentrations, plasma Aβ(1–40) and Aβ(1–42) levels, and the PK/PD relationship between them, successfully described the observed data, including the non-dose-proportional increase in plasma Aβ levels. This model suggested that reductions in free Aβ levels associated with crenezumab dosing are better maintained at higher doses, even after the total plasma Aβ levels had plateaued (Fig. 8).

Our analysis suggested that age and GFR explain some of the BSV in baseline Aβ levels. This is consistent with a previous study by Toledo and colleagues [18] who analyzed baseline Aβ measures in 715 subjects from the Alzheimer’s Disease Neuroimaging Initiative (ADNI) database (http://www.adni-info.org/index) and reported that age, platelet count, total protein, and creatinine concentration were independent predictors of baseline Aβ(1–40) and Aβ(1–42) levels, explaining 12.1% and 12.9% of the observed variability in the respective parameters [18]. The model estimated *K*_d_ of crenezumab against Aβ(1–40) and Aβ(1–42) were similar to each other. This is consistent with in vitro observation that crenezumab has similar binding affinity to these two Aβ species (~ 10 nM) [F. Hoffmann-La Roche Ltd.; data on file]. Patient factors, such as age or sex, were not identified as significant covariates for *K*_d_, suggesting that the binding of crenezumab to Aβ is independent of currently evaluated patient factors.

The PK/PD data from this study provide evidence of peripheral target engagement by crenezumab at evaluated dose levels. This complements the target engagement in the central nervous system previously suggested in the phase II ABBY and BLAZE studies through the increase of total monomeric Aβ(1–42) in CSF of patients with mild-to-moderate AD treated with crenezumab [8]. The increase in total monomeric Aβ(1–42) is likely due to slower elimination of crenezumab–Aβ complex. We further evaluated relationships between crenezumab concentration and Aβ(1–42) increase in CSF, but no clear correlation was observed (Fig. 5), which could be due to limited ranges of evaluable doses and high variability between patients for demonstrating exposure–response relationships in CSF. The concentration of crenezumab in CSF was much higher than the concentration of Aβ, yet still much lower than in serum (approximately 0.3% of serum). This suggests that a wider dose range is needed to be able to quantify the exposure–response relationship in the central nervous system.

The PK/PD simulation provided several important insights. Firstly, the simulation showed that circulating crenezumab is predominantly unbound. This indicates that the transfer of crenezumab to peripheral organs and the central nervous system is likely not influenced by its binding to Aβ. The low concentration of crenezumab–Aβ complex, compared with total crenezumab, also suggests that the transfer of the binding complex is unlikely to serve as a new source of Aβ for peripheral organs including the central nervous system.

Another interesting observation is that predicted free Aβ levels continued to decrease with increasing dose, even after the accumulation of total Aβ reached a plateau (Fig. 8), suggesting that total Aβ change may not be fully reflective of actual drug effect. The reason for this apparent discrepancy between total and free Aβ profiles is that accumulation of total Aβ is due to slower elimination of crenezumab–Aβ complex than free Aβ. Therefore, once the crenezumab–Aβ complex becomes the predominant species of total Aβ, there will be no further increase in total Aβ with increased crenezumab dose. In contrast, binding of crenezumab to residual free Aβ can still occur with a higher free crenezumab concentration. Additionally, since analytical quantification of free Aβ is technically challenging due to changing levels of bound vs free Aβ in vitro after CSF collection that might differ from the levels in vivo, the model proposed in the current study could be a promising tool to integrate available information, such as total Aβ levels, and provide further insights on the kinetics of unmeasured species such as free Aβ.

## Conclusion

In this study, we showed that crenezumab PK was dose proportional at doses between 15 and 120 mg/kg with a *t*_1/2_ consistent with IgG mAbs, and provided evidence of peripheral target engagement in patients with mild-to-moderate AD, based on data pooled from three clinical trials of crenezumab (two phase II studies; one phase Ib study). We also observed dose-dependent increases in total monomeric Aβ(1–42) levels in CSF; however, exposure–response relationships need to be further evaluated at higher doses and in larger studies. We demonstrated how a TMDD model can be used to integrate available information, such as the serum PK characteristics of crenezumab and the plasma total Aβ levels, and to make inferences to unmeasured species such as free Aβ levels.

## Data Availability

Qualified researchers may request access to individual patient-level data through the clinical study data request platform (www.clinicalstudydatarequest.com). Further details on Roche’s criteria for eligible studies are available here: https://clinicalstudydatarequest.com/Study-Sponsors/Study-Sponsors-Roche.aspx. For further details on Roche’s Global Policy on the Sharing of Clinical Information and how to request access to related clinical study documents, see here: https://www.roche.com/research_and_development/who_we_are_how_we_work/clinical_trials/our_commitment_to_data_sharing.htm.

## Abbreviations

A_Aβ_: Amount of beta-amyloid
AD: Alzheimer’s disease
ADNI: Alzheimer’s Disease Neuroimaging Initiative
AUC: Area under the curve
Aβ: Beta-amyloid
BSV: Between-subject variability
BWT: Body weight
*C*_cren_: Concentration of crenezumab
CL_el_: Elimination clearance
CL_int_: Intrinsic clearance
*C*_max_: Peak concentration
CSF: Cerebrospinal fluid
*C*_trough_: Pre-dose serum crenezumab
CV: Coefficient of variation
*F*_sc_: SC bioavailability
GFR: Glomerular filtration rate
IgG: Immunoglobulin G
IV: Intravenous
*K*_a_: Absorption rate constant
*K*_d_: Estimated equilibrium constant governing antibody-ligand binding
*K*_deg_: Degradation rate constant
*k*_int_: Zero-order input rate constant for ligand
mAb: Monoclonal antibody
OLE: Open-label extension
PD: Pharmacodynamics
PET: Positron emission tomography
PK: Pharmacokinetics
*Q*: Inter-compartment clearance
q2w: Every 2 weeks
q4w: Every 4 weeks
RSE: Relative standard error
SC: Subcutaneous
SCM: Stepwise covariate model
SD: Standard deviation
SRI: Safety run-in
*t*_1/2_: Half-life
TMDD: Target-mediated drug disposition
*V*_cent_: Central volume of distribution
*V*_periph_: Peripheral distribution volume
